# Larvicidal activity of lignans and alkaloid identified in *Zanthoxylum piperitum* bark toward insecticide-susceptible and wild *Culex pipiens pallens* and *Aedes aegypti*

**DOI:** 10.1186/s13071-017-2154-0

**Published:** 2017-05-04

**Authors:** Soon-Il Kim, Young-Joon Ahn

**Affiliations:** 1 NARESO R&D Center, Seoul National University Business Incubator, Suwon, 16614 South Korea; 2 Department of Agricultural Biotechnology, Seoul National University, Seoul, 08826 South Korea

**Keywords:** Botanical mosquito larvicide, *Zanthoxylum piperitum*, Rutaceae, Lignans, Xanthoxylol-γ,γ-dimethylallylether, Alkaloid, Insecticide resistance

## Abstract

**Background:**

The yellow fever mosquito, *Aedes aegypti*, and the common house mosquito, *Culex pipiens pallens*, transmit dengue fever and West Nile virus diseases, respectively. This study was conducted to determine the toxicity of the three lignans (–)-asarinin, sesamin and (+)-xanthoxylol-γ,γ-dimethylallylether (XDA), and the alkaloid pellitorine from *Zanthoxylum piperitum* (Rutaceae) bark to third-instar larvae from insecticide-susceptible *C. pipiens pallens* and *Ae. aegypti* as well as wild *C. pipiens pallens* resistant to deltamethrin, cyfluthrin, fenthion, and temephos.

**Methods:**

The toxicities of all isolates were compared with those of mosquito larvicide temephos. LC_50_ values for each species and their treatments were significantly different from one another when their 95% confidence intervals did not overlap.

**Results:**

XDA was isolated from *Z. piperitum* as a new larvicidal principle. XDA (LC_50_, 0.27 and 0.24 mg/l) was 4, 53, and 144 times and 4, 100, and 117 times more toxic than pellitorine, sesamin, and asarinin toward larvae from susceptible *C. pipiens pallens* and *Ae. aegypti*, respectively. Overall, all the isolates were less toxic than temephos (LC_50_, 0.006 and 0.009 mg/l). These constituents did not differ in toxicity to larvae from the two *Culex* strains. The present finding indicates that the lignans and alkaloid and the insecticides do not share a common mode of larvicidal action or elicit cross-resistance.

**Conclusion:**

Naturally occurring *Z. piperitum* bark-derived compounds, particularly XDA, merit further study as potential mosquito larval control agents or as lead compounds for the control of insecticide-resistant mosquito populations.

**Electronic supplementary material:**

The online version of this article (doi:10.1186/s13071-017-2154-0) contains supplementary material, which is available to authorized users.

## Background

The yellow fever mosquito, *Aedes aegypti* (Linnaeus, 1762) [1], and the common house mosquito, *Culex pipiens pallens* (Coquillett, 1898) [2], are found in tropical and subtropical regions of the world [3] and Eastern Asia [4], respectively, and are serious disease vectoring insect pests [5, 6]. A recent study calculated that more than 2.5 billion people are at risk of dengue infection over 100 countries worldwide, and there may be 50–100 million dengue infections annually, including 22,000 deaths every year, mostly among children [7]. From 1999 to 2015, 43,937 cases of human West Nile virus disease (including 20,265 neuroinvasive disease cases) were reported in the United States (US), which resulted in 1,911 deaths [8]. The most serious problem with the mosquito species is their ability to evolve resistance to insecticides rapidly [9]. Increasing levels of resistance to the conventional insecticides have resulted in multiple treatments and excessive doses, raising serious environmental and human health concerns. Widespread insecticide resistance has been one of the major obstacles in the cost-effective integrated vector management program. In addition, the number of approved insecticides may be reduced soon in the US by the US Environmental Protection Agency as reregistration occurs [10]. Reregistration requirement is also a concern in other regions including in the European Union, where it is under the control of the Commission Regulation (EC) No 1048/2005 [11]. Therefore, there is a high need for the development of selective control alternatives with novel target sites to establish a biorational resistance management strategy based on all available information on the extent and nature of resistance in mosquitoes because vaccines have limited effectiveness in controlling dengue [12].

Biocides derived from plants have been suggested as potential alternatives for mosquito control largely because plants constitute a potential source of bioactive secondary metabolites that are perceived by the public as relatively safe and with less risk to the environment, and with minimal impacts to human and animal health [13–19]. Phytochemicals act at multiple, novel target sites [14, 16–21], thereby reducing the potential for resistance [17–19, 22, 23]. Based on these benefits of botanical insecticides, numerous papers are published annually [19, 24]. Phytochemicals are regarded as potential sources to develop commercial mosquito larvicides as products derived from certain plants and their constituents meet the criteria as reduced risk insecticides [16–19, 25]. Recently, *Zanthoxylum* plants (Rutaceae) have drawn attention because they contain insecticidal constituents toward the cowpea aphid, *Aphis craccivora* Koch, 1854 [26, 27], the maize weevil, *Sitophilus zeamais* (Motschulsky, 1855) [28, 29], and larvae of various mosquito vectors [17, 30]. However, no previous studies have investigated the potential use of Japanese pepper, *Zanthoxylum piperitum* (L.) DC., for managing mosquitoes, particularly insecticide-resistant mosquitoes, despite its repellency to *Ae. aegypti* [31] and the stable fly, *Stomoxys calcitrans* (Linnaeus, 1758) [32, 33].

In this study, our aim was to assess whether the three lignans, asarinin, xanthoxylol-γ,γ-dimethylallylether (XDA) and sesamin, and the isobutylamide alkaloid pellitorine, extracted from the bark of *Z. piperitum*, had the toxicity to third-instar larvae from insecticide-susceptible *C. pipiens pallens* and *Ae. aegypti*, as well as wild colonies of *C. pipiens pallens* resistant to various insecticides [23]. The toxicity of the bark constituents was compared with that of the currently available mosquito larvicide temephos to assess their use as future commercial mosquito larvicides because it is registered as a larvicide for the control of mosquitoes in South Korea [34]. Also, the quantitative structure-activity relationship (QSAR) of the test compounds is discussed.

## Methods

### Instrumental analysis

The ^1^H and ^13^C nuclear magnetic resonance (NMR) spectra were recorded in CDCl_3_ on Varian NMR system spectrometers (Varian, Palo Alto, CA, USA), using tetramethylsilane as an internal standard. The chemical shifts are given in δ (ppm). The ultraviolet (UV) spectra were obtained in methanol on a UVICON 933/934 spectrophotometer (Kontron, Milan, Italy) and the mass spectra on a GSX 400 spectrometer (Jeol, Tokyo, Japan). Silica gel 60 (0.063–0.2 mm) (Merck, Darmstadt, Germany) and Sephadex LH-20 (Sigma-Aldrich, St. Louis, MO, USA) were used for column chromatography. Merck precoated silica gel plates (Kieselgel 60 F_254_) were used for analytical thin-layer chromatography (TLC). An Agilent 1200 series high-performance liquid chromatograph (Agilent, Santa Clara, CA, USA) was used to isolate the active constituents.

### Materials

The organophosphorus (OP) insecticide temephos (97.3%) was purchased from Riedel (Seelze, Lower Saxony, Germany). Triton X-100 was purchased from Coseal (Seoul, South Korea). All of the other chemicals used in this study were of reagent-grade quality and are available commercially.

### Mosquitoes

The stock cultures of *C. pipiens pallens* (susceptible KS-CP strain) and *Ae. aegypti* have been maintained in the laboratory without exposure to any known insecticide, as described previously [35]. Larvae from YS-CP colony of *C. pipiens pallens*, originally collected near rice paddy fields and cowsheds in Yusung (Daejeon, South Korea) in September 2010, showed extremely high levels of resistance to fenthion (resistance ratio (RR), 390) and deltamethrin (RR, 164) and moderate levels of resistance to cyfluthrin (RR, 14) and temephos (RR, 14) [23]. Adult mosquitoes were maintained on a 10% sucrose solution and blood fed on live mice. Larvae were reared in plastic trays (24 × 35 × 5 cm) containing 0.5 g of sterilised diet (40-mesh chick chow powder/yeast, 4/1 by weight). All stages were held at 27 ± 1 °C, 65–75% relative humidity, and a 14:10 h light:dark cycle.

### Plant material

Fresh bark of *Z. piperitum* was collected from the Southern Forest Resources Research Center (Jinju, Gyeongnam, South Korea), National Institute of Forest Science, in mid-August 2009. A certified botanical taxonomist was used to identify the plant. A voucher specimen (ZP-01) was deposited in the Research Institute of Agriculture and Life Sciences, College of Agriculture and Life Sciences, Seoul National University.

### Extraction and isolation

Air-dried bark (550 g) of *Z. piperitum* was pulverised, extracted with methanol (3.3 L) two times at room temperature for 2 days, and filtered. The combined filtrate was concentrated to dryness by rotary evaporation at 40 °C to yield approximately 70 g of a dark brownish sticky solid. The extract (20 g) was sequentially partitioned into hexane- (6.4 g), chloroform- (1.36 g), ethyl acetate- (0.46 g), and water-soluble (11.78 g) portions for the subsequent bioassays. This fractionation procedure was repeated three times. The organic solvent-soluble portions were concentrated under vacuum at 35 °C, and the water-soluble portion was freeze-dried. To isolate the active constituents, 10–50 mg/l of each *Z. piperitum* bark-derived fraction was tested in a mortality bioassay, as described by Perumalsamy et al. [22].

**Fig. 1:**
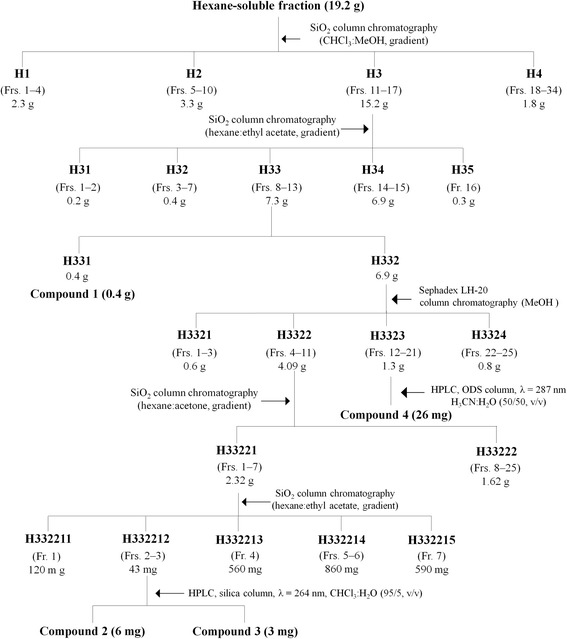
Procedures to isolate the mosquito larvicidal constituents. The *Zanthoxylum piperitum* bark methanol extract was sequentially partitioned into hexane-, chloroform-, ethyl acetate-, and water-soluble portions. The hexane-soluble fraction was the most biologically active fraction, and HPLC was performed. Each fraction (10–50 mg/l) was tested in a mortality bioassay to isolate the active constituents from the fraction

The hexane-soluble fraction (19.2 g) was the most biologically active fraction (Table 1) and was chromatographed on a 5.5 × 70 cm silica gel (500 g) column by elution with a gradient of chloroform and methanol [(100:0 (2 l), 95:5 (1 l), 90:10 (2 l), 80:20 (1 l), 50:50 (1 l), and 0:100 (1.5 l) by volume] to provide 34 fractions (each approximately 250 ml) (Fig. 1). The column fractions were monitored by TLC on silica gel plates developed with a chloroform and methanol (9:1 by volume) mobile phase. Column fractions with similar *R*_f_ values on the TLC plates were pooled. The spots were detected by spraying the plate with 4% H_2_SO_4_ and then heating on a hot plate. Active fractions 11–17 (H3) were pooled and rechromatographed on a 5.5 × 70 cm silica gel (500 g) column by elution with a gradient of hexane and ethylacetate [(90:10 (1 l), 80:20 (1 l), and 0:100 (1 l) by volume] and finally with 1 l methanol to afford 16 fractions (each approximately 250 ml). The fractions were monitored by TLC on silica gel plates developed with a hexane and ethyl acetate (7:3 by volume) mobile phase. Active fractions 8–13 (H33) were pooled and crystallised during being dried by rotary evaporation at 35 °C to yield compound one (H331). The residual portion (H332) was isolated by Sephadex LH-20 column chromatography using a mobile phase of methanol. Two active fractions 4–11 (H3322) and 12–21 (H3323) were obtained. The H3322 fraction (4.09 g) was rechromatographed on a 5.5 × 70 cm silica gel (120 g) column. Separation was achieved with a gradient of hexane and acetone [80:20 (2 l), 70:30 (1 l), 50:50 (1 l), and 0:100 (0.5 l) by volume] and finally with 1 l methanol to afford 25 fractions (each approximately 200 ml). Column fractions were monitored by TLC on silica gel plates developed with a hexane and ethyl acetate (4:6 by volume) mobile phase. Active fractions 1–7 (H33221) were obtained. Fraction H33221 was rechromatographed on a silica gel column using a gradient of chloroform and ethyl acetate [20:1 (0.3 l), 10:1 (0.2 l), 8:2 (0.2 l), 1:1 (0.1 l), and 0:10 (0.5 l) by volume] and finally with acetone (0.2 l) to afford five fractions (each approximately 200 ml). A preparative high-performance liquid chromatography (HPLC) was performed to separate the constituents from the active H332212 fraction. The column was a 3.9 mm i.d. × 300 mm bondaclone ten silica (Phenomenex, Torrance, CA, USA) using a mobile phase of chloroform and ethyl acetate (95:5 by volume) at a flow rate of 1 ml/min. Chromatographic separation was monitored using a UV detector at 264 nm. The two active constituents two and three were isolated at retention times of 8.05 and 10.03 min, respectively. For separation of a constituent from another active H3323 fraction (1.3 g), a preparative HPLC was performed. The column was a 21.2 mm i.d. × 250 mm Phenomenex Prodigy ODS with a mobile phase of acetonitrile and water (1:1 by volume) at a flow rate of 1 ml/min. Chromatographic separation was monitored at 287 nm. Finally, an active constituent four was isolated at a retention time of 5.35 min.

**Table 1:** Toxicity of fractions obtained from solvent partitioning of methanol extract of *Zanthoxylum piperitum* bark to third-instar larvae from *Culex pipiens pallens* during a 24 h exposure

Material	*n* ^a^	Slope ± SE	LC_50_, mg/l (95% CI^b^)	LC_90_, mg/l (95% CI^b^)	*χ* ^2c^	*P*-value
Methanol extract	240	5.1 ± 0.57	5.91 (5.38–6.44)	10.50 (9.28–12.54)	3.25	0.974
Hexane-soluble fraction	240	4.3 ± 0.47	4.18 (3.69–4.64)	8.27 (7.26–9.89)	4.90	0.932
Chloroform-soluble fraction	240	5.1 ± 0.54	5.02 (4.53–5.50)	9.02 (8.04–10.58)	6.01	0.921
Ethyl acetate-soluble fraction	60		>100			
Water-soluble fraction	60		> 100			

^a^Number of larvae tested

^b^CI denotes confidence interval

^c^Pearson’s chi-square goodness-of-fit test

### Bioassay

A mortality bioassay [36] was used to assess the toxicity of all compounds to third-instar larvae from the susceptible and wild mosquitoes. In brief, each compound in acetone was suspended in distilled water with Triton X-100 (20 μl/l). Groups of 20 mosquito larvae were separately put into paper cups (270 ml) containing each compound solution (250 ml). Temephos served as a positive control and was similarly formulated. Negative controls consisted of the acetone-Triton X-100 solution in distilled water. Based on the preliminary test results, the toxicity of each test compound and insecticide was determined with four to six concentrations ranging from 0.1 to 100 mg/l and 0.001 to 0.1 mg/l, respectively. All treatments were replicated three times using 20 larvae per replicate.

Treated and control (acetone-Triton X-100 solution only) larvae were held under the same conditions as those used for colony maintenance without providing food. Larval mortalities were determined 24 h post-treatment. A larva was considered dead if it did not move when prodded with a fine wooden dowel [22].

### Data analysis

Data were corrected for control mortality using Abbott’s formula [37]. Concentration-mortality data were subjected to probit analysis [38]. A compound having LC_50_ > 100 mg/l was ineffective as described by Kiran et al. [39]. The LC_50_ values for each species and their treatments were significantly different from one another when their 95% confidence intervals did not overlap.

## Results

### Bioassay-guided fractionation and isolation

The fractions obtained from the solvent partitioning of the methanol extract of the *Z. piperitum* bark were bioassayed toward third-instar larvae from insecticide-susceptible *C. pipiens pallens* (Table 1) and *Ae. aegypti* (Table 2). Significant differences in toxicity were observed among the fractions and were used to identify the peak activity fractions for the next step of purification. Based on the 24 h LC_50_ values, the hexane-soluble fraction was the most toxic material, followed by the chloroform-soluble fraction. No toxicity was obtained using the ethyl acetate- or water-soluble fractions. Mortality in the acetone-Triton X-100-water-treated controls for any the species in this study was less than 2%.

**Table 2:** Toxicity of fractions obtained from solvent partitioning of methanol extract of *Zanthoxylum piperitum* bark to third-instar larvae from *Aedes aegypti* during a 24 h exposure

Material	*n* ^a^	Slope ± SE	LC_50_, mg/l (95% CI^b^)	LC_90_, mg/l (95% CI^b^)	*χ* ^2c^	*P*-value
Methanol extract	240	4.3 ± 0.47	3.95 (3.47–4.40)	7.88 (6.92–9.40)	4.03	0.945
Hexane-soluble fraction	240	4.0 ± 0.43	4.21 (3.75–4.73)	8.78 (7.47–11.19)	3.90	0.951
Chloroform-soluble fraction	240	3.8 ± 0.47	5.68 (5.06–6.33)	12.30 (10.37–15.94)	4.14	0.941
Ethyl acetate-soluble fraction	60		> 100			
Water-soluble fraction	60		> 100			

^a^Number of larvae tested

^b^CI denotes confidence interval

^c^Pearson’s chi-square goodness-of-fit test

**Fig. 2:**
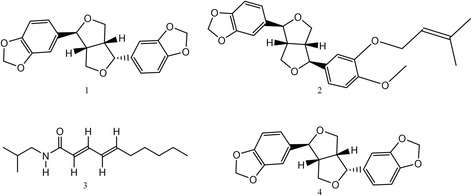
Structures of asarinin, xanthoxylol-γ,γ-dimethylallylether, pellitorine, and sesamin. These compounds were identified in the bark of *Zanthoxylum piperitum* in this study. The chemical formula of (–)-asarinin (1) is C_20_H_18_O_6_, with a molar mass of 354.35 g/mol; the chemical formula of (+)-xanthoxylol-γ,γ-dimethylallylether (2) is C_25_H_28_O_6_, with a molar mass of 424.48 g/mol; the chemical formula of pellitorine (3) is C_14_H_25_NO, with a molar mass of 223.35 g/mol; and the chemical formula of sesamin (4) is C_20_H_18_O_6_, with a molar mass of 354.35 g/mol

Bioassay-guided fractionation of the *Z. piperitum* bark extract afforded four active compounds that were identified by spectroscopic analyses, including electron ionized mass spectrometry (EI-MS) and NMR spectroscopy. The four active compounds were (–)-asarinin (5-[3-(1,3-benzodioxol-5-yl)-1,3,3a,4,6,6a-hexahydrofuro[3,4-c]furan-6-yl]-1,3-benzodioxole) (1), (+)-xanthoxylol-γ,γ-dimethylallylether (XDA) (2), pellitorine [(2*E*,4*E*)-*N*-(2-methylpropyl)deca-2,4-dienamide] (3), and sesamin [5,5′-(1*S*,3a*R*,4*S*,6a*R*)-tetrahydro-1*H*,3*H*-furo[3,4-*c*]furan-1,4-diylbis(1,3-benzodioxole)] (4) (Fig. 2). (–)-Asarinin (1) was identified based on the following evidence: white powder. EI-MS (70 eV), *m/z* (% relative intensity): 354 [M]^+^, 336, 203, 161, 149, 135, 122 (Additional file 1). ^1^H NMR (CDCl_3_, 500 MHz): δ 2.85 (1H, dd, *J* = 7.0, 14.0 Hz), 3.30 (1H, m), 3.83 (2H, m), 3.83–4.09 (2H, m), 4.40 (1H, d, *J* = 9.5 Hz), 4.82 (1H, d, *J* = 7.0 Hz), 5.95 (4H, d, *J* = 6.0 Hz), 6.79 (4H, m), 6.86 (2H, s) (Additional file 2). ^13^C NMR (CDCl_3_, 125 MHz): δ 50.1 t, 54.6 t, 69.6 d, 70.9 d, 82.0 d, 87.6 d, 100.9 t, 101.0 t, 106.3 d, 106.5 d, 108.1 d, 118.6 d, 119.5 d, 132.2 s, 135.1 s, 146.5 s, 147.1 s, 147.6 s, 147.9 s (Additional file 3). (+)-Xanthoxylol-γ,γ-dimethylallylether (2) was characterized as follows: viscous solid. EI-MS (70 eV), *m/z* (% relative intensity): 424 [M]^+^, 356 (100), 325, 205, 178, 149, 135, 69 (Additional file 4). ^1^H NMR (CDCl_3_, 600 MHz): δ 1.74 (3H, s), 1.78 (3H, s), 2.92 (1H, q), 3.34 (1H, m), 3.86 (3H, m), 3.88 (3H, s), 4.12 (1H, d, *J* = 9.0 Hz), 4.42 (1H, d, *J* =6.6 Hz), 4.59 (2H, d, *J* = 6.6 Hz), 4.85 (1H, d, *J* = 5.4 Hz), 5.53 (1H, m), 5.97 (2H, s), 6.78 (1H, m), 6.81 (1H, m), 6.84 (1H, m), 6.86 (1H, m), 6.87 (1H, m), 6.92 (1H, d, *J* = 1.2 Hz) (Additional file 5). ^13^C NMR (CDCl_3_, 150 MHz): δ 18.4 q, 26.0 q, 50.4 d, 54.7 d, 56.1 t, 66.1 t, 69.9 d, 71.2 d, 82.3 d, 87.9 d, 101.2 q, 106.6 d, 108.4 d, 109.6 d, 113.2 d, 118.6 d, 118.9 d, 120.2 d, 132.5 s, 133.8 s, 137.8 s, 146.8 s, 147.5 s, 147.9 s, 149.9 s (Additional file 6). Pellitorine (3) was characterized as follows: viscous oil. EI-MS (70 eV), *m/z* (% relative intensity): 223 [M]^+^, 208, 180, 167, 152 (100), 113, 96, 72 (Additional file 7). ^1^H NMR (CDCl_3_, 400 MHz): ^1^H NMR (CDCl_3_, 400 MHz): δ 0.88 (3H, s), 0.91 (3H, s), 0.93 (3H, s), 1.28 (4H, m), 1.37 (2H, m), 1.76 (1H, m), 2.13 (2H, dd, *J* = 7.0, 13.8 Hz), 3.16 (2H, t, *J* = 6.4, 12.9 Hz), 5.60 (1H, br s), 5.76 (1H, d, *J* = 15.0 Hz), 6.09 (2H, m), 7.19 (1H, d, *J* = 15.0 Hz) (Additional file 8). ^13^C NMR (CDCl_3_, 100 MHz): δ 14.0 q, 20.1 q, 22.5 t, 28.5 t, 28.6 d, 31.4 t, 32.9 t, 46.9 t, 121.7 d, 128.2 d, 128.2 d, 141.2 d, 166.4 s (Additional file 9). Sesamin (4) was characterized as follows: colorless crystals. EI-MS (70 ev), *m/z* (% relative intensity): 354 [M]^+^, 323, 203, 178, 161, 149 (100), 135 (Additional file 10). ^1^H NMR (CDCl_3_, 500 MHz): δ 3.05 (2H, m), 3.86 (2H, dd, *J* = 3.0, 9.0 Hz), 4.23 (2H, dd, *J* = 6.5, 9.0 Hz), 4.71 (2H, d, *J* = 4.0 Hz), 5.95 (4H, s), 6.79 (4H, d, *J* = 8.0 Hz), 6.85 (2H, s) (Additional file 11). ^13^C NMR (CDCl_3_, 125 MHz): δ 50.1 t, 54.6 t, 69.6 d, 70.9 d, 82.0 d, 87.6 d, 100.9 t, 101.0 t, 106.3 d, 106.5 d, 108.1 d, 118.6 d, 119.5 d, 122.0 s, 132.2 s, 146.5 s, 147.1 s, 147.6 s, 147.9 s (Additional file 12).

### Larvicidal activity of test compounds

The toxicity of the four isolated constituents to third-instar larvae from KS-CP strain of *C. pipiens pallens* was likewise compared with that of temephos, which was used as a positive control (Table 3). Responses varied according to compound examined. Based on the 24 h LC_50_ values, XDA (0.27 mg/l) was the most toxic compound, followed by pellitorine (1.12 mg/l). These constituents were 45 and 187 times less toxic than temephos, respectively. LC_50_ of sesamin was 14.28 mg/l. The toxicity of asarinin was the lowest of any of the compounds examined. Interestingly, the toxicity of all constituents was virtually identical toward both insecticide-susceptible and wild third-instar *C. pipiens pallens* larvae (Table 4), indicating a lack of cross-resistance in the resistant larvae.

**Table 3:** Toxicity of *Zanthoxylum piperitum* bark constituents and temephos to third-instar larvae from insecticide-susceptible KS-CP strain of *Culex pipiens pallens* during a 24 h exposure

Compound	*n* ^a^	Slope ± SE	LC_50_, mg/l (95% CI^b^)	LC_90_, mg/l (95% CI^b^)	*χ* ^2c^	*P*-value
XDA (2)^d^	420	1.8 ± 0.10	0.27 (0.24–0.30)	1.44 (1.21–1.79)	6.61	0.980
Pellitorine (3)	300	2.5 ± 0.23	1.12 (0.95–1.35)	3.75 (2.95–5.20)	5.67	0.957
Sesamin (4)	240	2.8 ± 0.31	14.28 (12.24–16.66)	40.48 (32.03–56.93)	1.82	0.997
Asarinin (1)	300	4.4 ± 0.45	38.90 (35.26–42.50)	75.77 (67.01–89.69)	6.34	0.932
Temephos	300	1.4 ± 0.18	0.006 (0.005–0.008)	0.049 (0.032–0.096)	2.49	0.999

^a^Number of larvae tested

^b^CI denotes confidence interval

^c^Pearson’s chi-square goodness-of-fit test

^d^Xanthoxylol-γ,γ-dimethylallylether

**Table 4:** Toxicity of *Zanthoxylum piperitum* bark constituents and temephos to third-instar larvae from wild YS-CP colony of *Culex pipiens pallens* during a 24 h exposure

Compound	*n* ^a^	Slope ± SE	LC_50_, mg/l (95% CI^b^)	LC_90_, mg/l (95% CI^b^)	*χ* ^2c^	*P*-value
XDA (2)^d^	300	1.9 ± 0.21	0.31 (0.26–0.38)	1.39 (1.03–2.13)	6.23	0.937
Pellitorine (3)	300	2.1 ± 0.29	1.42 (1.17–1.80)	5.46 (3.71–10.29)	1.81	0.997
Sesamin (4)	300	2.3 ± 0.24	12.64 (10.48–14.90)	45.73 (36.14–63.54)	5.96	0.947
Asarinin (1)	240	5.5 ± 0.67	33.80 (31.30–36.47)	57.67 (51.01–69.59)	4.41	0.927
Temephos	360	4.1 ± 0.34	0.149 (0.133–0.166)	0.307 (0.271–0.358)	6.90	0.975

^a^Number of larvae tested

^b^CI denotes confidence interval

^c^Pearson’s chi-square goodness-of-fit test

^d^Xanthoxylol-γ,γ-dimethylallylether

Toward third-instar *Ae. aegypti* larvae (Table 5), XDA (LC_50_, 0.24 mg/l) was the most toxic compound, followed by pellitorine (LC_50_, 0.98 mg/l), as judged by the 24 h LC_50_ values. These constituents were 27 and 109 times less toxic than temephos, respectively. LC_50_ of sesamin and asarinin was 23.98 and 28.15 mg/l, respectively.

**Table 5:** Toxicity of *Zanthoxylum piperitum* bark constituents and temephos to third-instar larvae of *Aedes aegypti* during a 24 h exposure

Compound	*n* ^a^	Slope ± SE	LC_50_, mg/l (95% CI^b^)	LC_90_, mg/l (95% CI^b^)	*χ* ^2c^	*P*-value
XDA (2)^d^	300	1.8 ± 0.17	0.24 (0.20–0.30)	1.29 (0.95–1.97)	6.37	0.983
Pellitorine (3)	240	2.6 ± 0.30	0.98 (0.84–1.16)	2.98 (2.30–4.36)	3.49	0.967
Sesamin (4)	240	2.4 ± 0.25	23.98 (20.48–28.33)	82.75 (63.03–122.72)	2.66	0.998
Asarinin (1)	300	7.5 ± 0.79	28.15 (26.25–29.95)	41.60 (38.51–46.18)	2.07	0.995
Temephos	240	1.6 ± 0.38	0.009 (0.007–0.012)	0.062 (0.032–0.373)	1.48	0.999

^a^Number of larvae tested

^b^CI denotes confidence interval

^c^Pearson’s chi-square goodness-of-fit test

^d^Xanthoxylol-γ,γ-dimethylallylether

## Discussion

Certain plant-derived materials and their constituents can be developed into products suitable for integrated mosquito management because they are selective, biodegrade to nontoxic products, have few harmful effects on nontarget organisms, are environmentally nonpersistent, and can be used in conjunction with biological control [13–19, 40]. These potential mosquito larvicides can be applied to mosquito breeding places in the same manner as conventional mosquito larvicides. Although several plant preparations meet the criteria for efficacy, only a very few commercial botanical products have been marketed because there are some formidable barriers to commercialization, as well described previously [17–19, 40]. In addition, some botanical insecticides may contain nonselective substances that may have a negative impact on small nontarget organisms such as crustaceans and zooplankton [17, 40] and human (e.g. contact dermatitis, allergic reactions, severe acute poisoning) [40]. Many plant-derived products and their constituents manifest toxicity to different mosquito species larvae [13, 14, 16, 17, 30], and have been proposed as alternatives to conventional mosquito larvicides. Komalamisra et al. [41] considered larvicidal products exerting LC_50_ < 50 mg/l active, 50 mg/l < LC_50_ < 100 mg/l moderately active, 100 mg/l < LC_50_ < 750 mg/l effective, and LC_50_ > 750 mg/l inactive. Kiran et al. [39] considered compounds with LC_50_ < 100 mg/l as exhibiting a significant larvicidal effect. It has been reported that the most promising botanical mosquito control agents are plants in the families Asteraceae, Cladophoraceae, Lamiaceae, Meliaceae, Oocystaceae, and Rutaceae [13]. The efficacy of various plant extracts and their fractions (LC_50_, 2.6–44,400 mg/l) and essential oils (LC_50_, 0.2–194 mg/l; LC_90_, 0.5–260 mg/l) toward various mosquito species larvae has been well documented by Shaalan et al. [14] and Pavela [17], respectively, although the larvicidal activity can vary significantly according to plant species, chemotypes, plant tissue, age of plant, geographic conditions, solvent used in extraction, and mosquito species [13, 17, 40]. In the current study, *Z. piperitum* (Rutaceae) bark methanol extract and its hexane- and chloroform-soluble fractions exhibited good larvicidal activity toward *C. pipiens pallens* (LC_50_, 4.18–5.91 mg/l; LC_90_, 8.27–10.50 mg/l) and *Ae. aegypti* (LC_50_, 3.95–5.68 mg/l; LC_90_, 7.88–12.30 mg/l). *Zanthoxylum piperitum* is distributed in northeast Asia (Korea, China, and Japan) [42], and the *Z. piperitum* bark contains unsaturated aliphatic acid amides [43]. Pavela and Govindarajan [30] reported that the *Zanthoxylum monophyllum* leaf essential oil had potent larvicidal activity toward *Anopheles subpictus* (LC_50_ and LC_90_, 41.50 and 82.19 mg/l), *Aedes albopictus* (LC_50_ and LC_90_, 45.35 and 88.07 mg/l), and *Culex tritaeniorhynchus* (LC_50_ and LC_90_, 49.01 and 92.08 mg/l).

Phytochemicals such as alkaloids, phenols and terpenoids, alone or in combination, contribute to acute toxicity toward various arthropod species [15]. Active larvicidal constituents (LC_50_ < 50 mg/l) [41] derived from plants include alkaloids (e.g. pellitorine, guineensine, pipercide, and retrofractamide A, LC_50_ 0.004–0.86 mg/l [44]), terpenoids (e.g. quassin, LC_50_ 6.0 mg/l [45]; germacrene D-4-ol and α-cadinol, LC_50_ 6.12–7.26 and 10.27–12.28 mg/l [30]), coumarins (e.g. imperatorin and osthole, LC_50_ 2.88 and 3.14 mg/l [23]), flavonoids (e.g. karanjin, karanjachromene, pongamol, and pongarotene, LC_50_ 14.61–37.61 mg/l [46]), phenylpropanoids (e.g. methyleugenol and α-asarone, LC_50_ 10.49 and 26.99 mg/l [22]; ethyl *p*-methoxycinnamate and ethyl cinnamate, LC_50_ 12.3 and 24.1 mg/l [36]), neolignans (e.g. conocarpan, eupomatenoid-5, and eupomatenoid-6, LC_50_ < 10 mg/l [47]), cyanogenic glycosides (e.g. dhurrin, LC_50_ 1.12 mg/l [48]), lactones (e.g. goniothalmin, LC_50_ 0.87–25.95 mg/l [49]), acetylenic alcohols (e.g. falcarinol and falcarindiol, LC_50_ 3.49 and 6.51 mg/l [50]), phenols (e.g. 4-butoxymethylphenol, LC_50_ 0.05 mg/l [51]), and fatty acids (e.g. oleic acid and palmitic acid, 18.07–18.45 and 34.50–42.96 mg/l [46]).

In the current study, we used a mortality bioassay to identify the larvicidal constituents from the *Z. piperitum* bark extracts. The active constituents were determined to be the furofuranoid lignans (–)-asarinin (1), (+)-XDA (2) and sesamin (4), and the isobutylamide alkaloid pellitorine (3). The interpretations of the proton and carbon signals of compounds 1, 2, 3, and 4 were largely consistent with those of Perumalsamy et al. [22], Biavatti et al. [52], Perumalsamy et al. [22] and Park et al. [44], and Ju et al. [53], respectively. XDA was isolated from *Z. piperitum* as a new larvicidal constituent. This compound was most toxic toward larvae of two vector mosquito species, although it was less toxic than temephos. Pellitorine was also highly toxic toward *C. pipiens pallens* and *Ae. aegypti,* as described previously [22, 45]. In addition, these constituents were also effective toward *C. pipiens pallens* larvae resistant to various insecticides. The present finding indicates that *Z. piperitum* bark-derived preparations containing the active constituents, particularly XDA and pellitorine, hold promise for the development of novel, effective, naturally occurring mosquito larvicides even toward currently insecticide-resistant mosquito populations, because XDA (LC_50_ 0.24–0.27 mg/l for two mosquito species) and pellitorine (LC_50_ 0.98–1.12 mg/l for two mosquito species) meet the stage 3 criteria (LC_50_ < 1 mg/l) set by Shaalan et al. [14]. The next step stage 4 involves the determination of effective field application rates of various formulations in simulated field trials and/or small-scale field trials [14].

QSARs of phytochemicals in many insects have been well noted. For example, Wang et al. [23] studied the toxicity of six linear furanocoumarins including imperatorin and six simple coumarins including osthole. They reported that the chemical structure and alkoxy substitution and length of the alkoxyl side chain at the C8 position are essential for imparting toxicity. Park et al. [44] reported that the larvicidal activity toward three vector mosquito species was much more pronounced in compounds such as guineensine, pipercide, and retrofractamide A with an isobutylamine moiety than in one such as piperine without this moiety among the methylenedioxyphenyl (MDP)-containing compounds. In addition, the isobutylamides with an MDP moiety was more active than the ones without an MDP moiety. The MDP moiety is thought to stabilise the chemical structure [54]. In the current study, XDA with an MDP moiety was more toxic than either asarinin or sesamin with two MDP moieties. In addition, sesamin was more toxic than asarinin, 7-epimer of sesamin. Our findings, along with previous studies, indicate that other factor(s) such as chemical structure, functional group, and isomerism, as well as hydrophobic (log *P*) and molecular refraction parameters, may play, in part, a role in determining the lignan toxicities to mosquito larvae, although the MDP moiety might contribute, to some extent, to the larvicidal effect.

An investigation of the modes of action and the resistance mechanisms of biolarvicides may contribute to the development of selective mosquito control alternatives with novel target sites. Major mechanisms of resistance to insecticides currently available to control mosquitoes are target site insensitivity that reduces sodium channel sensitivity to pyrethroid insecticides or sensitivity of acetylcholinesterase to OP and carbamate insecticides, as well as enhanced metabolism of various groups of insecticides [55, 56]. Some phytochemicals were found to be highly effective toward insecticide-resistant mosquitoes [14, 22, 23], and they are likely to be useful in resistance management strategies. For example, imperatorin and osthole are effective toward larvae from wild *C. pipiens pallens* with extremely high to moderate levels of resistance to cyfluthrin, deltamethrin, fenthion, and temephos [22]. The current findings that the three furofuranoid lignans and the isobutylamide alkaloid described were of equal toxicity to both insecticide-susceptible and -resistant larvae of *C. pipiens pallens* suggest that the phytochemicals and the pyrethroid and OP insecticides do not share a common mode of action or elicit cross-resistance. Detailed tests are needed to understand fully the exact mode of action of the furofuranoid lignans and the isobutylamide alkaloid, although the octopaminergic and γ-aminobutyric acid receptors have been suggested as novel target sites for some monoterpenoid essential oil constituents in the American cockroach [57] and the cotton bollworm [20] and the fruit fly [21], respectively. It has also been reported that tannins and pellitorine primarily affect the midgut epithelium and secondarily affect the gastric caeca and the malpigian tubules in *C. pipiens* larvae [58] and *Ae. aegypti* larvae [59], respectively.

## Conclusion

*Zanthoxylum piperitum* bark-derived products containing xanthoxylol-γ,γ-dimethylallylether and pellitorine could be useful as larvicides in the control of mosquito populations, particularly in the light of their activity toward insecticide-resistant mosquito larvae. Further research is needed on the practical applications of plant-derived preparations as novel mosquito larvicides to establish their safety profiles in humans, although *Z. piperitum* is commonly used as a spice and as a traditional medicinal plant [60, 61]. In addition, their effects on nontarget aquatic organisms including larvivorous fishes, biological control agents for mosquitoes [62], and the aquatic environment need to be established. Lastly, detailed tests are needed to understand how to improve the larvicidal potency and stability of the compounds isolated from *Z. piperitum* for eventual commercial development.

## Additional files

Additional file 1: EI-MS spectrum of (–)-asarinin (1). (TIF 115 kb)

Additional file 2: ^1^H NMR (CDCl_3_, 500 MHz) spectrum of (–)-asarinin (1). (TIF 60 kb)

Additional file 3: ^13^C NMR (CDCl_3_, 125 MHz) spectrum of (–)-asarinin (1). (TIF 62 kb)

Additional file 4: EI-MS spectrum of (+)-xanthoxylol-γ,γ-dimethylallylether (2). (TIF 167 kb)

Additional file 5: ^1^H NMR (CDCl_3_, 600 MHz) spectrum of (+)-xanthoxylol-γ,γ-dimethylallylether (2). (TIF 98 kb)

Additional file 6: ^13^C NMR (CDCl_3_, 150 MHz) spectrum of (+)-xanthoxylol-γ,γ-dimethylallylether (2). (TIF 87 kb)

Additional file 7: EI-MS spectrum of pellitorine (3). (TIF 80 kb)

Additional file 8: ^1^H NMR (CDCl_3_, 400 MHz) spectrum of pellitorine (3). (TIF 110 kb)

Additional file 9: ^13^C NMR (CDCl_3_, 100 MHz) spectrum of pellitorine (3). (TIF 82 kb)

Additional file 10: EI-MS spectrum of sesamin (4). (TIF 63 kb)

Additional file 11: ^1^H NMR (CDCl_3_, 500 MHz) spectrum of sesamin (4). (TIF 62 kb)

Additional file 12: ^13^C NMR (CDCl_3_, 125 MHz) spectrum of sesamin (4). (TIF 42 kb)

## Abbreviations

EI-MS: Electron ionized mass spectrometry
HPLC: High-performance liquid chromatography
MDP: Methylenedioxyphenyl
NMR: Nuclear magnetic resonance
OP: Organophosphorus
QSAR: Quantitative structure-activity relationship
RR: Resistance ratio
TLC: Thin-layer chromatography
UV: Ultra violet
XDA: (+)-xanthoxylol-γ,γ-dimethylallylether
