# Supplementary figures and images for: Larvicidal activity of lignans and alkaloid identified in Zanthoxylum piperitum bark toward insecticide-susceptible and wild Culex pipiens pallens and Aedes aegypti
Source: Parasit Vectors. 2017 May 4;10:221. doi: 10.1186/s13071-017-2154-0 (PMC5418860; doi:10.1186/s13071-017-2154-0)

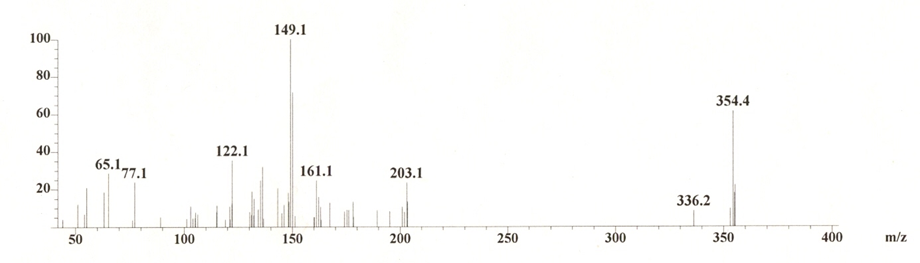

Supplement: Supplementary file 1 — EI-MS spectrum of (–)-asarinin (1). (TIF 115 kb) [file 13071_2017_2154_MOESM1_ESM.tif]

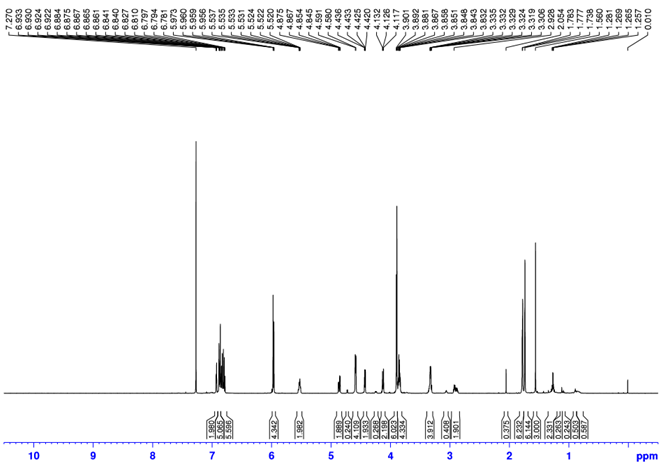

Supplement: Supplementary file 2 — 1H NMR (CDCl3, 500 MHz) spectrum of (–)-asarinin (1). (TIF 60 kb) [file 13071_2017_2154_MOESM2_ESM.tif]

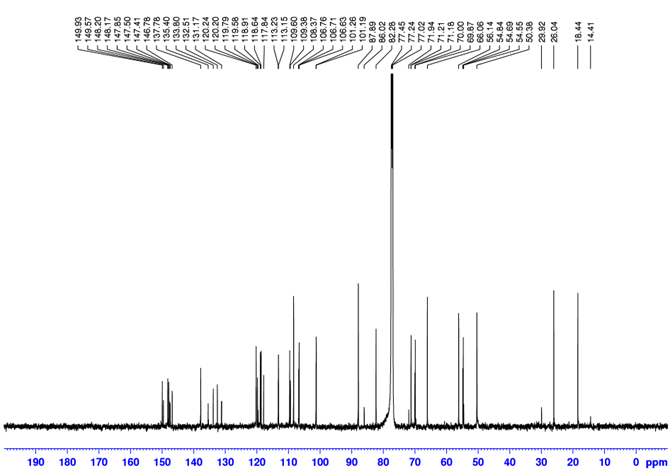

Supplement: Supplementary file 3 — 13C NMR (CDCl3, 125 MHz) spectrum of (–)-asarinin (1). (TIF 62 kb) [file 13071_2017_2154_MOESM3_ESM.tif]

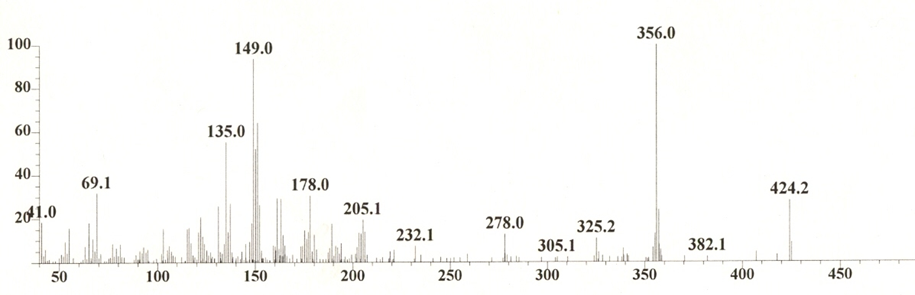

Supplement: Supplementary file 4 — EI-MS spectrum of (+)-xanthoxylol-γ,γ-dimethylallylether (2). (TIF 167 kb) [file 13071_2017_2154_MOESM4_ESM.tif]

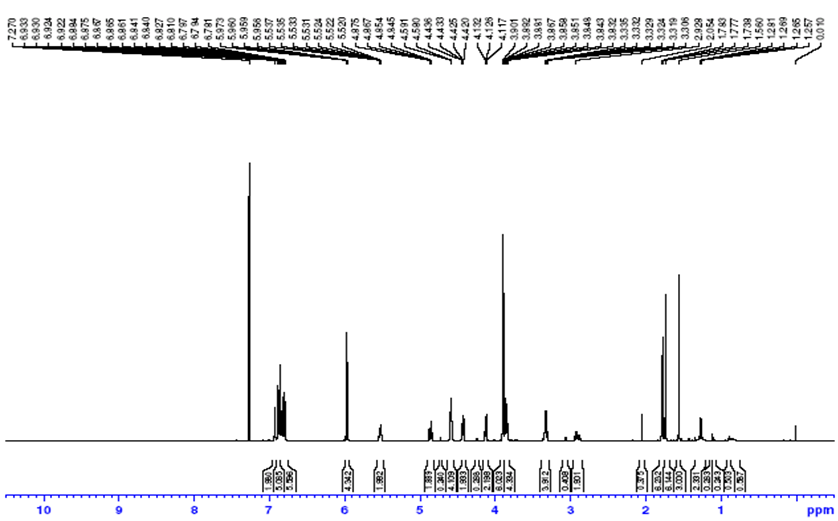

Supplement: Supplementary file 5 — 1H NMR (CDCl3, 600 MHz) spectrum of (+)-xanthoxylol-γ,γ-dimethylallylether (2). (TIF 98 kb) [file 13071_2017_2154_MOESM5_ESM.tif]

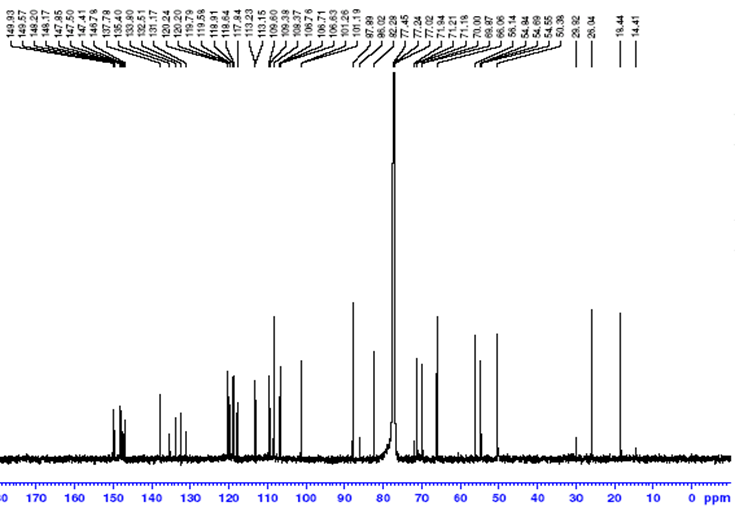

Supplement: Supplementary file 6 — 13C NMR (CDCl3, 150 MHz) spectrum of (+)-xanthoxylol-γ,γ-dimethylallylether (2). (TIF 87 kb) [file 13071_2017_2154_MOESM6_ESM.tif]

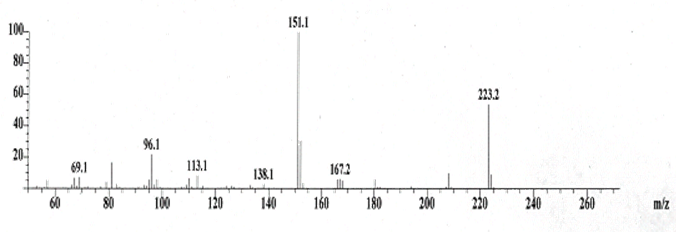

Supplement: Supplementary file 7 — EI-MS spectrum of pellitorine (3). (TIF 80 kb) [file 13071_2017_2154_MOESM7_ESM.tif]

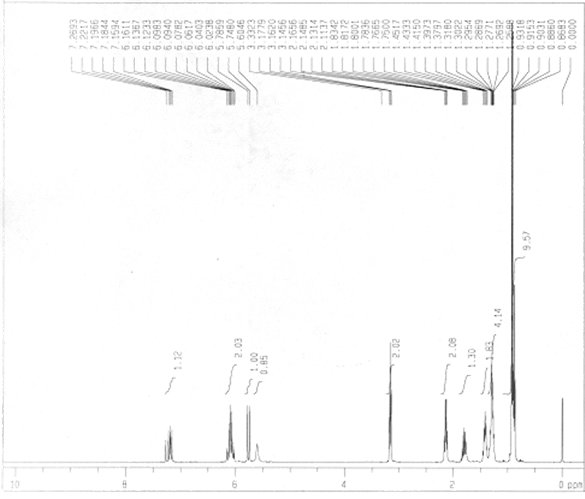

Supplement: Supplementary file 8 — 1H NMR (CDCl3, 400 MHz) spectrum of pellitorine (3). (TIF 110 kb) [file 13071_2017_2154_MOESM8_ESM.tif]

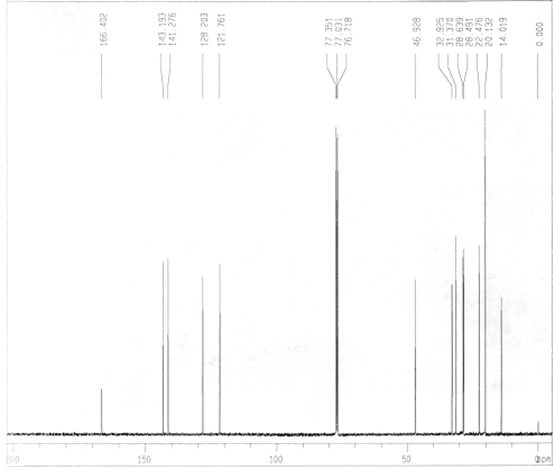

Supplement: Supplementary file 9 — 13C NMR (CDCl3, 100 MHz) spectrum of pellitorine (3). (TIF 82 kb) [file 13071_2017_2154_MOESM9_ESM.tif]

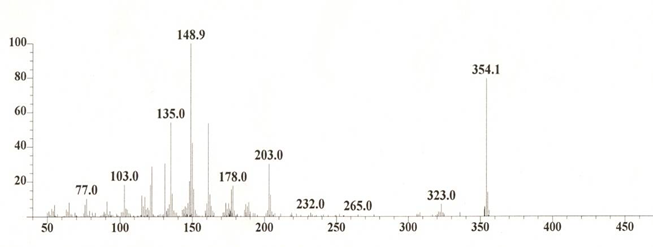

Supplement: Supplementary file 10 — EI-MS spectrum of sesamin (4). (TIF 63 kb) [file 13071_2017_2154_MOESM10_ESM.tif]

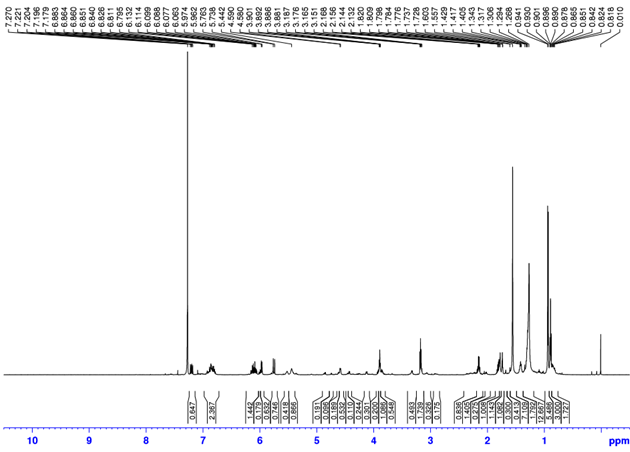

Supplement: Supplementary file 11 — 1H NMR (CDCl3, 500 MHz) spectrum of sesamin (4). (TIF 62 kb) [file 13071_2017_2154_MOESM11_ESM.tif]

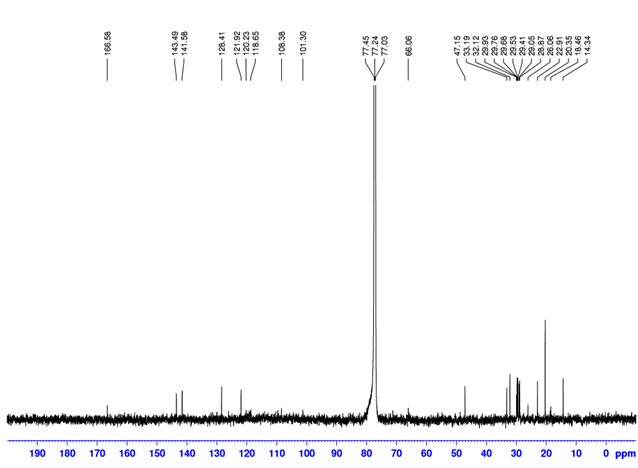

Supplement: Supplementary file 12 — 13C NMR (CDCl3, 125 MHz) spectrum of sesamin (4). (TIF 42 kb) [file 13071_2017_2154_MOESM12_ESM.tif]
